# A novel YGGT family protein is localized in the apicoplast and is essential for the organelle inheritance

**DOI:** 10.3389/fcimb.2025.1642716

**Published:** 2025-08-04

**Authors:** Wenqiang Su, Haorong Gu, Jun Zheng, Honglin Jia

**Affiliations:** State Key Laboratory for Animal Disease Control and Prevention, Harbin Veterinary Research Institute, Chinese Academy of Agricultural Sciences (CAAS), Harbin, China

**Keywords:** *Toxoplasma gondii*, apicoplast, biogenesis, YGGT family protein, *Tg*YCAP

## Abstract

*Toxoplasma gondii* is an obligate intracellular apicomplexan parasite. Most apicomplexan parasites contain an endosymbiont-derived organelle called the apicoplast. This organelle is critical for the survival of parasites because it plays a role in several essential metabolic pathways. However, the molecular mechanisms involved in maintaining the apicoplast have not been well understood. In this study, we investigated the function of an apicoplast-residing protein called *Tg*YCAP in the inheritance of the apicoplast. Our results showed that conditional knockdown of *Tg*YCAP severely inhibited the growth of the parasite and disrupted the inheritance of the apicoplast. In addition, the YGGT domain is essential for its function in the apicoplast.

## Introduction

*Toxoplasma gondii* is a type of Apicomplexa protozoa that is widely distributed worldwide. It can infect most warm-blooded animals, including humans. Infections of *T. gondii* do not cause symptoms in most people. However, such infections are more likely to cause severe disease and even death in patients suffering from AIDS or who have undergone organ transplantation.

*T. gondii* possesses a unique organelle called the apicoplast, which is believed to have originated from a secondary endosymbiotic event. The apicoplast is an isolated organelle surrounded by four membranes. The outermost membrane likely originates from the endomembrane system. The second outer membrane, called the periplastid membrane (PPM), has an unclear origin but is thought to derive from the plasma membrane of red algae. The two innermost membranes of the organelle are believed to have evolved from the plastid of red algae (Zhang et al., 2021). Although the apicoplast does not play a role in photosynthesis, similar to non-green plastids, it is essential for several basic metabolic pathways critical for the survival of parasites in various host environments (van Dooren et al., 2008). Consequently, these pathways are of great interest for research.

The molecular mechanisms behind the biogenesis of the apicoplast are not yet fully understood. The apicoplast has a highly reduced genome, with most proteins expressed by the parasite’s genome needing to be transported to the apicoplast. It is known that an endoplasmic reticulum-associated degradation (ERAD) system, referred to as SELMA, is likely located on the peripheral plasma membrane (PPM) of the apicoplast in *T. gondii*. Additionally, translocon complexes, known as TIC-TOC, are thought to localize to the inner two membranes of the apicoplast to facilitate intra-apicoplast transport. However, many of the subunits of this complex remain missing or unidentified. Furthermore, the apicoplast undergoes elongation and division during the budding process of daughter parasites. Several proteins involved in the division of the apicoplast has been identified, including the dynamin-related protein TgDrpA (van Dooren et al., 2009), F-actin, the actin polymerization protein FRM2 (Tosetti et al., 2019), and myosin F (MyoF) (Devarakonda et al., 2023). Nevertheless, the exact mechanisms of apicoplast division have not yet been fully elucidated.

YGGT family proteins are found exclusively in bacteria and eukaryotes that contain plastids (Kabeya et al., 2010), but their function remains unclear. Photosynthetic eukaryotes can have up to four YGGT members, all of which are predicted to reside in the chloroplasts (Kabeya et al., 2010). In both chloroplasts and cyanobacteria, YGGT proteins are thought to be associated with the distribution of nucleoids (Kabeya et al., 2010). Additionally, the ylmG gene is part of the division and cell wall (dcw) gene cluster, located downstream of the cell division gene ftsZ in bacterial genomes (Fadda et al., 2003). Therefore, YlmG is suggested to play a role in cell division. However, YlmG was identified as part of the TIC complex and is suggested to play a role in the formation of the preprotein translocation pathway in another study (Jin et al., 2022; Liang et al., 2024).

In contrast, the functions of YGGT family proteins from apicomplexan parasites is completely unknown. In this study, we report that a protein from the YGGT family, known as *Tg*YCAP, is localized within the apicoplast. The depletion of this protein impairs the lytic cycle of *T. gondii* and affects the inheritance of the apicoplast. Furthermore, we found that while the YGGT domain of *Tg*YCAP is not necessary for its localization to the apicoplast, it is essential for its functional role within the apicoplast.

## Materials and methods

### Bioinformatics analyses and identification of *Tg*YCAP

The *Tg*YCAP (ToxoDB: TGGT1_213100) protein sequence was obtained from the ToxoDB website (http://toxodb.org/toxo). The structural domain of the *Tg*YCAP protein was predicted using NCBI conserved domain search tool. Homologs of *Tg*YCAP in Apicomplexa were found by BLAST queries using EuPathDB (https://legacy.eupathdb.org/eupathdb/showApplication.do).

### Plasmids

PCR was performed using KOD-Plus-Neo (TOYOBO, # KOD-401B) or PrimeSTAR GXL DNA Polymerase (TaKaRa, # R050A). pCD-Cas9, which expresses the Cas9 gene C-terminally tagged with GFP and the ToxoU6 promoter to drive gRNA expression, was described previously (Zheng et al., 2016). The gRNA sequences targeting the cutting sites in *T. gondii* were designed by the EuPaGDT website (http://grna.ctegd.uga.edu/), according to previously reported methods (Brown et al., 2017). The target sequence of *Tg*YCAP with the gRNA scaffold was inserted into pCD-Cas9 at the *Pme*I site.

To construct the expression plasmids, total RNA was extracted from 4×10^7^ RH tachyzoites washed with phosphate-buffered saline (PBS) using a RNeasy Plus Mini Kit (Qiagen, # 74134) and reverse-transcribed into cDNA using a One-Step RT-PCR Master Mix Kit (TOYOBO, # FSQ-201). *Tg*YCAP-EGFP, truncated *Tg*YCAP-EGFP, were amplified and inserted into the pDHFR-GRA or pHX-GRA vector at the *Eco*RV site under the control of the *Tg*GRA1 promoter.

To construct the plasmid to express *Tg*YCAP in *T. gondii*, the gRNA-targeting sequence of *Tg*YCAP was synonymously mutated. The open reading frame of *Tg*YCAP was C-terminally tagged with HA and then inserted into pTet-On-TAP at the *Xho*I site. pTet-On-TAP was a gift from David Sibley (Addgene plasmid # 59017; http://n2t.net/addgene:59017; RRID: Addgene_59017).

To analyze the function of the YGGT domain, *Tg*YCAP lacking this sequence was fused with a Flag tag at the C-terminus and cloned into the pDHFR-GRA vector (pDHFR-GRA-*TgYCAP*ΔYGGT-Flag).

### Antibodies

Anti-*Tg*SAG2, anti-*Tg*CPN60, and anti-*Tg*Tubulin sera were obtained by immunizing rabbits (Cao et al., 2020). A mouse anti-GFP mAb was purchased from Abbkine (# A02020). A rabbit anti-HA (# 3724S) antibody was purchased from CST. Mouse anti-HA (# H9658) and rabbit anti-FLAG (# 14793S) mAbs were from Cell Signaling Technology. A mouse anti-V5 mAb was from Invitrogen (#377500). Horseradish peroxidase-conjugated goat anti-mouse (# 115-035-003) and anti-rabbit (# 711-035-152) antibodies were purchased from Jackson. Goat anti-mouse IgG H&L Alexa Fluor^®^ 488 (# A11001) and 594 (# A11032) and goat anti-rabbit IgG H&L Alexa Fluor^®^ 488 (# A11034) and 594 (# A11037) antibodies were purchased from Invitrogen.

### Generation of conditional *Tg*YCAP-knockdown parasites

*T. gondii* parasites (RHΔhxgprt) were maintained in BJ-5ta cells (ATCC, # CRL-4001) at 37°C in 5% CO_2_. The growth medium was Dulbecco’s modified Eagle medium containing 10% fetal bovine serum (CLARK, #FB25015) and 50 μg ml^-1^ penicillin/streptomycin. *T. gondii* tachyzoites and all transgenic strains were cultured using standard procedures. The RHΔKu80 strain was obtained by deleting the Ku80 gene using the CRISPR/Cas9 method (Cao et al., 2020). The RHΔKu80 strain was transfected with the pCAT-YFP-TetR plasmid, which contains an *Escherichia coli* Tn10 tet-repressor (TetR), and selected using chloramphenicol (20 μM) (van Poppel et al., 2006) to generate the RHΔKu80::YFP-TetR strain. pCAT-YFP-TetR was a gift from David Sibley (Addgene plasmid # 59018; http://n2t.net/addgene:59018; RRID: Addgene_59018). The Tet-On-*Tg*YCAP-HA strain was obtained by transfecting the RHΔKu80::YFP-TetR strain with a plasmid expressing *Tg*YCAP-HA (cloned into the pTet-On-TAP vector) and selected using phleomycin (5 μg ml^-1^). The conditional knockdown strain was obtained by deleting the endogenous *Tg*YCAP gene based on the Tet-On-*Tg*YCAP-HA strain named as i*Tg*YCAP strain. For *TgYCAP*-knockdown experiments, Tc was added to a final concentration of 0.3 μg ml^-1^. To analyze the function of YGGT domain, conditional knockdown parasites were transfected with the pHX-GRA-*Tg*YCAP-ΔYGGT plasmid and selected using mycophenolic acid (25μg ml^-1^)/xanthine (50 μg ml^-1^).

### Parasite transfection

Parasites were transfected via electroporation as previously described (Soldati and Boothroyd, 1993). For each transfection, 10–20 million extracellular parasites in 400 μl CytoMix buffer were mixed with up to 20 μg purified plasmid and/or 5 μg amplicon DNA in a 2 mm electrode gap cuvette and electroporated with a MicroPulser Electroporator (Bio-Rad). The transfection conditions were 1600 V, 25 μF, and 50 Ω. Transfected parasites were grown in cell cultures for 24 h before drug selection or seeded into 96-well plates. The screened monoclonal strains were then confirmed via PCR, immunofluorescence analysis, and Western blotting.

### Immunofluorescence assays

Primary antibodies were used at a dilution of 1:1000, except the rabbit anti-*Tg*CPN60 antibody, which was used at a dilution of 1:500. Secondary Alexa Fluor 488- and 594-conjugated anti-mouse and anti-rabbit antibodies were used at a dilution of 1:1000. For general immunofluorescence microscopy of intracellular *T. gondii*, monolayers of human foreskin fibroblasts (HFFs) grown on glass-bottom cell culture dishes with a diameter of 20 mm (NEST, MH0031) were infected with *T. gondii* and then fixed in PBS containing 4% (w/v) paraformaldehyde for 30 min at room temperature. The samples were permeabilized with PBS containing 0.3% (v/v) Triton X-100 for 30 min, blocked with PBS containing 5% (w/v) bovine serum albumin, and then incubated with primary and secondary antibodies diluted in the blocking solution. Labeled parasites were washed three times with PBS and then observed using a Zeiss epifluorescence microscope.

### Western blotting

Extracellular parasites were harvested, washed with cold PBS, and lysed in RIPA buffer (Sigma, # R0278) on ice for 30 min. SDS-PAGE was performed under reducing conditions. A primary antibody and a horseradish peroxidase-conjugated goat anti-mouse or anti-rabbit secondary antibody diluted 1:3000 were used for Western blot analysis. Signals were visualized via enhanced chemiluminescence on blue-sensitive X-ray film (FUJI, # 4741023951) using a SuperSignal West Pico Chemiluminescent Substrate Kit (Thermo Fisher Scientific).

### Plaque assays

Monolayers of HFFs grown in 12-well plates were infected with 2500 tachyzoites per well. After incubation for 8 d in the presence or absence of Tc under normal growth conditions, the parasites were washed, fixed in PBS containing 4% (w/v) paraformaldehyde for 30 min, and stained with Giemsa solution (Solarbio, # G1015-500). One representative example from three independent experiments is presented.

### Statistical analysis

Results of the replication and plaque assays are expressed as mean values ± standard deviations. Statistical differences were determined using the paired or unpaired t-test with the Bonferroni test using GraphPad Prism 7.0. The significance of p-values was denoted as follows: ns, non-significant; *, *p* ≤ 0.05; **, *p* ≤ 0.01; ***, *p* ≤ 0.001; and ****, *p* ≤ 0.0001.

## Results

### A novel YGGT family protein localized in the apicoplast

**Figure 1:**
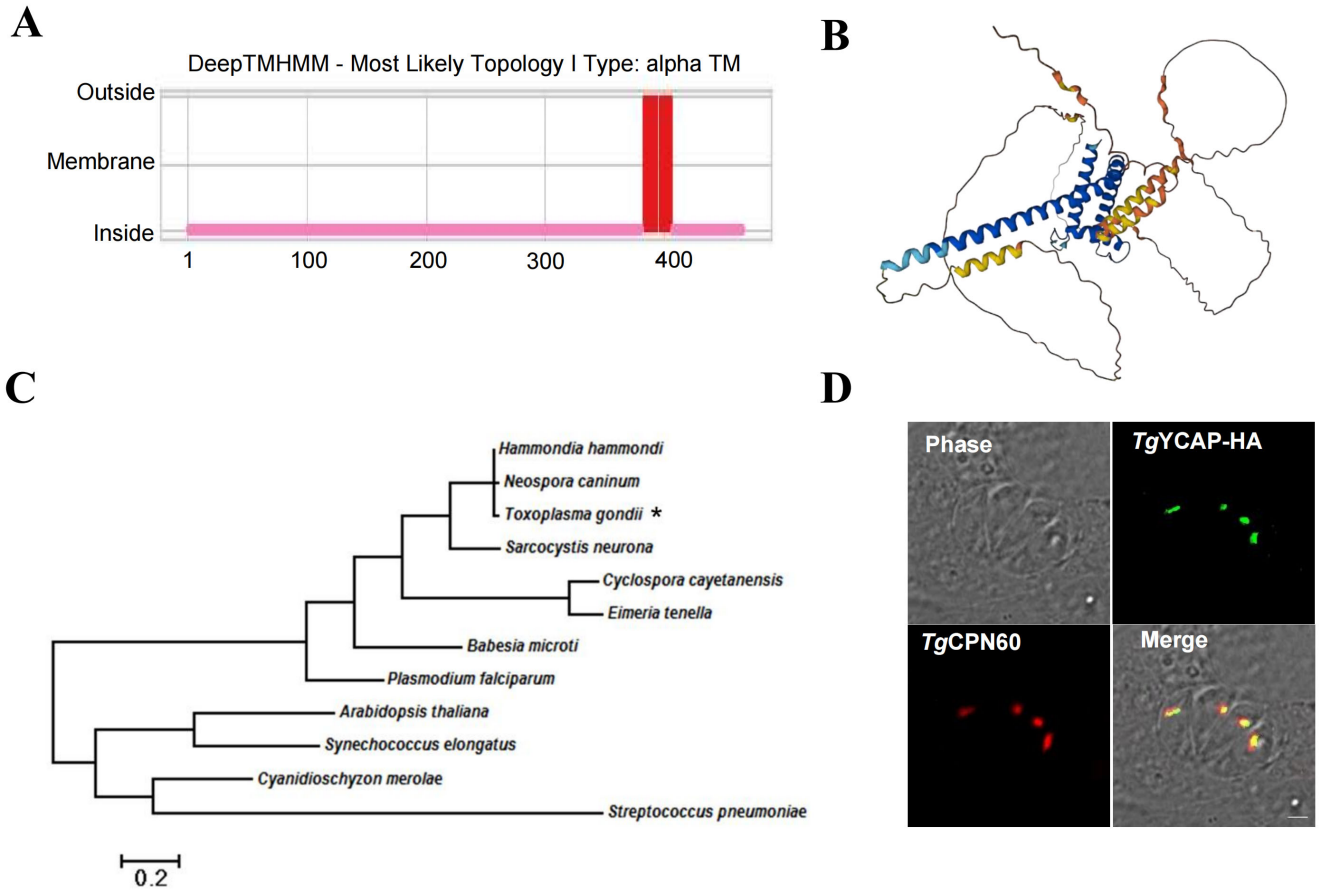
TgYCAP is localized in the apicoplast. **(A)** Prediction of transmembrane regions in TgYCAP using the DeepTMHMM algorithm (https://services.healthtech.dtu.dk/services/DeepTMHMM-1.0/). **(B)** Predicted structure of TgYCAPby. The three-dimensional structure was created with the AlphaFold Monomer v2.0 pipeline (https://alphafold.ebi.ac.uk/entry/S7UP54) (Jumper et al., 2021; Varadi et al., 2024). **(C)** Phylogenetic analysis of TgYCAP alongside its orthologues. TgYCAP is indicated by "*". **(D)** Parasites were transiently transfected with a plasmid expressing *Tg*YCAP fused with a HA tag (green), cultured for 24 h, and labeled with mouse anti-HA and rabbit anti-TgCPN60 antibodies (apicoplast marker, red).

A YGGT domain family protein (TGGT1_213100) was found by a screening conducted in our lab previously (unpublished). This protein was also mentioned in a recent report by Boucher et al. (2018), which utilized a proximity-based biotinylation approach to screen for novel proteins located in the parasitophorous vacuole (PV) and apicoplast, but its localization was not confirmed. The transmembrane topology predicted using the DeepTMHMM online tool indicates that this protein has two α-helices, with its N-terminal and C-terminal regions oriented within the membrane (**Figure 1A**). The structure predicted by AlphaFold shows that the C-terminal is composed almost entirely of α-helices (**Figure 1B**). Phylogenetic analysis shows that it was conserved among apicomplexan parasites, but it lacks a clear ortholog in Cryptosporidium spp. (**Figure 1C**). We have named this protein *Tg*YCAP (YGGT domain-containing Apicoplast Protein). The localization of *Tg*YCAP was examined by transiently transfecting a plasmid that expresses *Tg*YCAP fused with an HA tag. The results demonstrated that the fluorescence signal aligned well with the apicoplast marker TgCPN60, indicating that *Tg*YCAP is localized in the apicoplast (**Figure 1D**).

### Amino acids 1–200 of *Tg*YCAP are critical for its trafficking to the apicoplast

**Figure 2:**
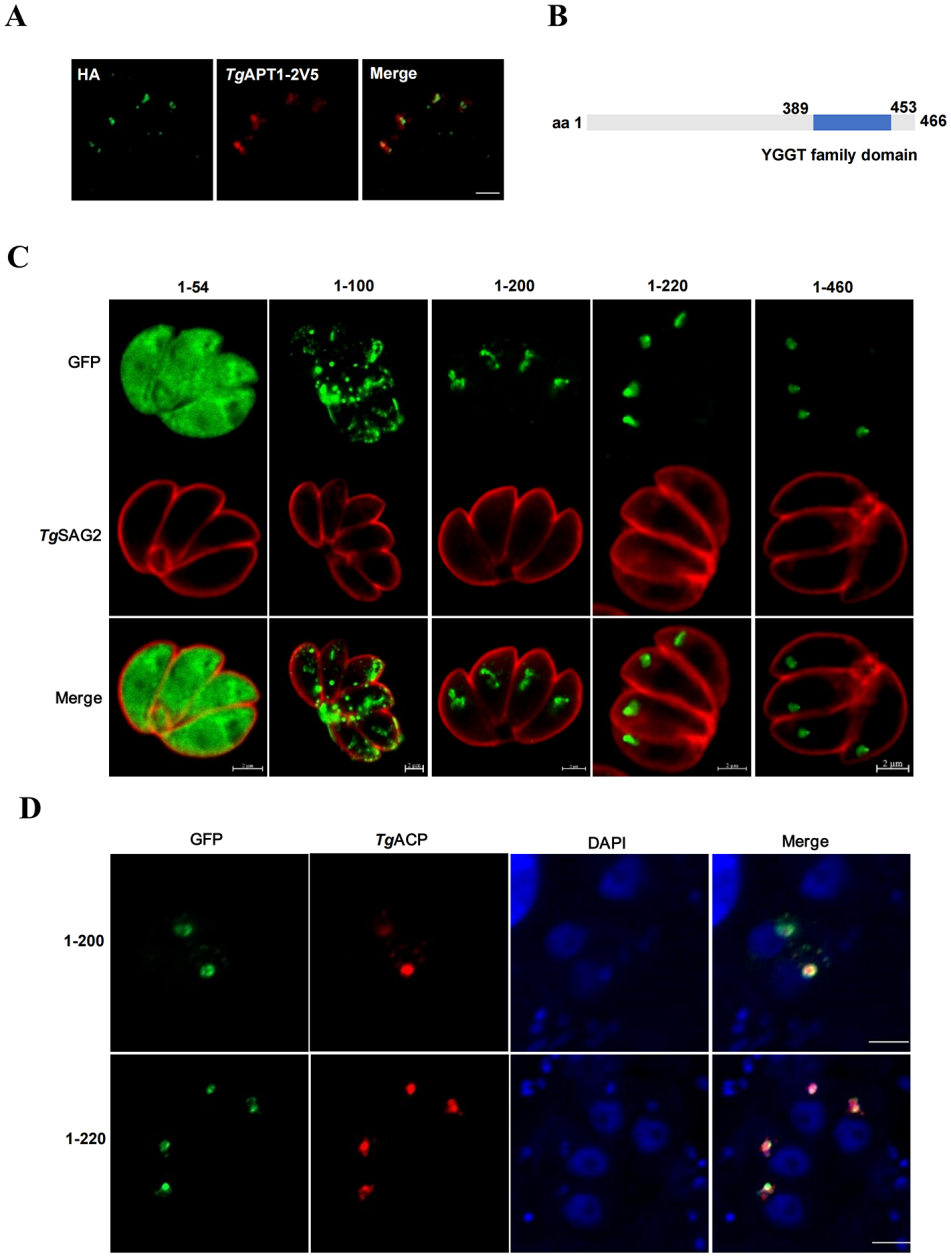
Amino acids 1–200 of *Tg*YCAP are involved in trafficking of this protein to the apicoplast. **(A)** The parasites stably expressing *Tg*YCAP-3×HA (green) were transiently transfected with a plasmid expressing *Tg*APT1-2V5 (red) and labeled with anti-HA and anti-V5 antibodies. **(B)** Diagram of *Tg*YCAP structure. The conserved domains were identified by Conserved Domain Search (https://www.ncbi.nlm.nih.gov/Structure/cdd/wrpsb.cgi). **(C)** Parasites transfected with the truncated *Tg*YCAP proteins fused with GFP were stained with a rabbit anti-*Tg*SAG2 antibody (red) and a mouse anti-GFP antibody (green). **(D)** Parasites co-transfected with plasmids expressing the truncated *Tg*YCAP proteins fused with GFP and a plasmid expressing TgACP fused with mcherry (red) were stained with a mouse anti-GFP antibody (green).

The localization of endogenous *Tg*YCAP to the apicoplast was confirmed by inserting a 3×HA tag at its C-terminal in the genomic locus by using CRISPR/Cas9 method. The proper insertion of the 3×HA tag was confirmed by a diagnostic PCR and sequencing analysis (**Supplementary Figure S1**). Then, we used an apicoplast-residing transporter, *Tg*APT1, fused with a 2×V5 tag (Fu et al., 2023) for co-localization analysis. The results showed that the fluorescence signal of *Tg*APT1 matched with that of *Tg*YCAP (**Figure 2A**). Bioinformatic analysis indicated that there is no clear signal peptide in *Tg*YCAP. Additionally, the YGGT domain was found to be localized close to the C-terminal by the Conserved Domain Search tool (**Figure 2B**). To examine the region that is responsible for the trafficking of *Tg*YCAP to the apicoplast, we fused a GFP tag to the C-terminal of several truncated versions of the protein. Indirect immunofluorescence analysis indicated that the region of the first 200 amino acids at the N-terminal of *Tg*YCAP, but not the YGGT domain is critical for its proper trafficking to the apicoplast (**Figures 2C, D**).

### *Tg*YCAP is a membrane-associated protein

**Figure 3:**
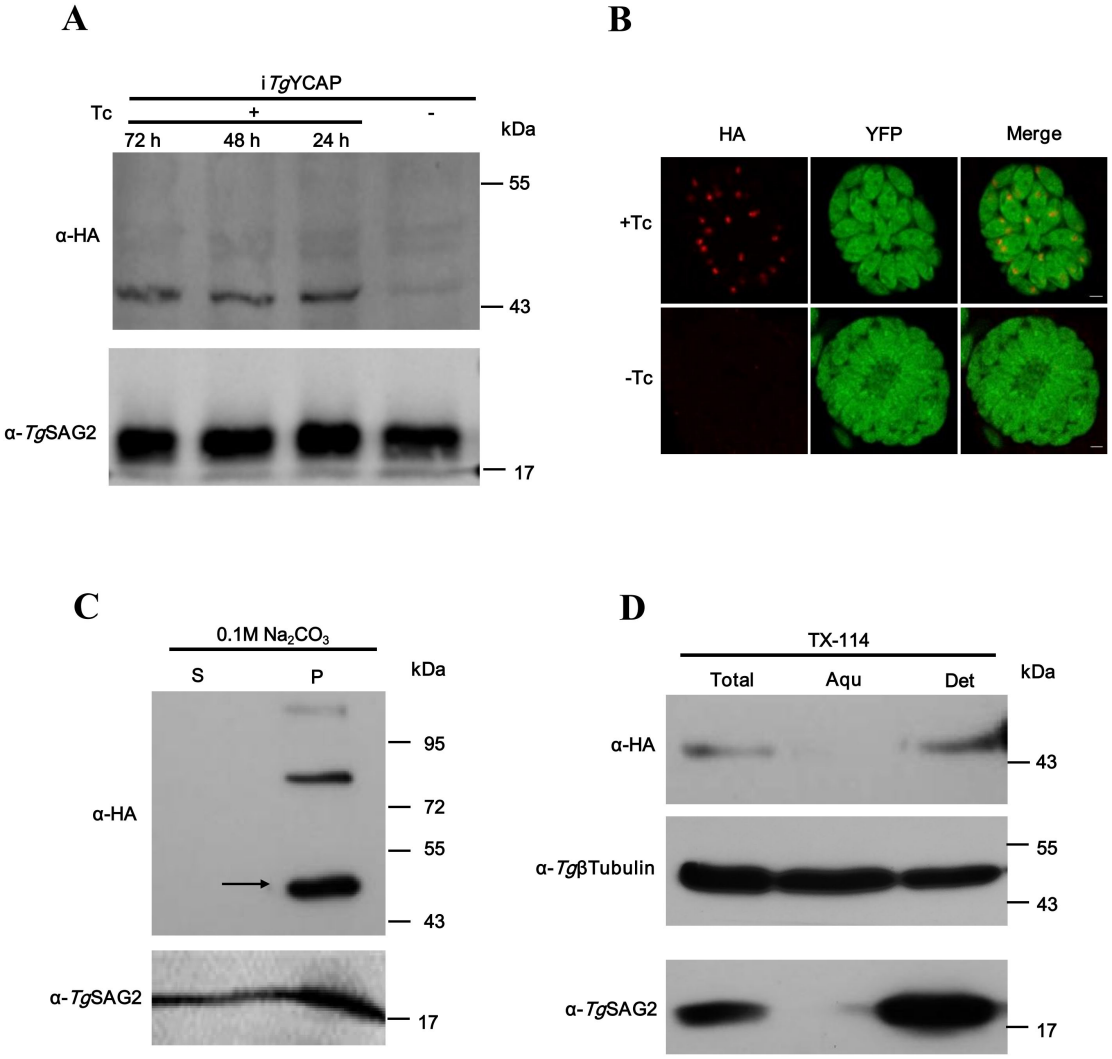
*Tg*YCAP is a membrane-associated protein at the apicoplast in *T. gondii.* **(A)** Western blot analysis was conducted to assess the expression of *Tg*YCAP fused with a HA tag, regulated by Tetracycline. **(B)** Immunofluorescence analysis was performed to examine *Tg*YCAP expression under Tetracycline regulation. *Tg*SAG2 was detected as a loading control. **(C)** Proteins were extracted from parasites and were then fractionated into soluble (S) and membrane pellet (P) fractions using sodium carbonate treatment. **(D)** Triton X-114 fractionation was carried out, revealing that *Tg*YCAP preferentially partitioned into the detergent (Det) phase, along with the soluble marker TgSAG2. In contrast, tubulin was found in either the aqueous (Aqu) phase or in the detergent fraction.

The expression level of *Tg*YCAP was extremely low. We could not detect any signals by Western blot analysis in the parasites, in which *Tg*YCAP was endogenously tagged with 3×HA at the C-terminus. To examine the membrane association of this protein, we cloned the open reading frame of *Tg*YCAP fused with a 3×HA tag into the pTet-On-TAP vector (Etheridge et al., 2014) and transfected it into RHΔKu80 parasites expressing a YFP-fused tetracycline repressor (YFP-TetR) (van Poppel et al., 2006). Parasites were selected using phleomycin. In these parasites, expression of *Tg*YCAP was easily detected in the presence of tetracycline (Tc) by Western blotting (**Figure 3A**) and IFAT (**Figure 3B**). The parasite lysate was treated with Na_2_CO_3_ and Triton X-114 to analyze the membrane association of *Tg*YCAP. The results revealed that *Tg*YCAP is a membrane-associated protein (**Figures 3C, D**).

### *Tg*YCAP is required for the survival of *T. gondii*

**Figure 4:**
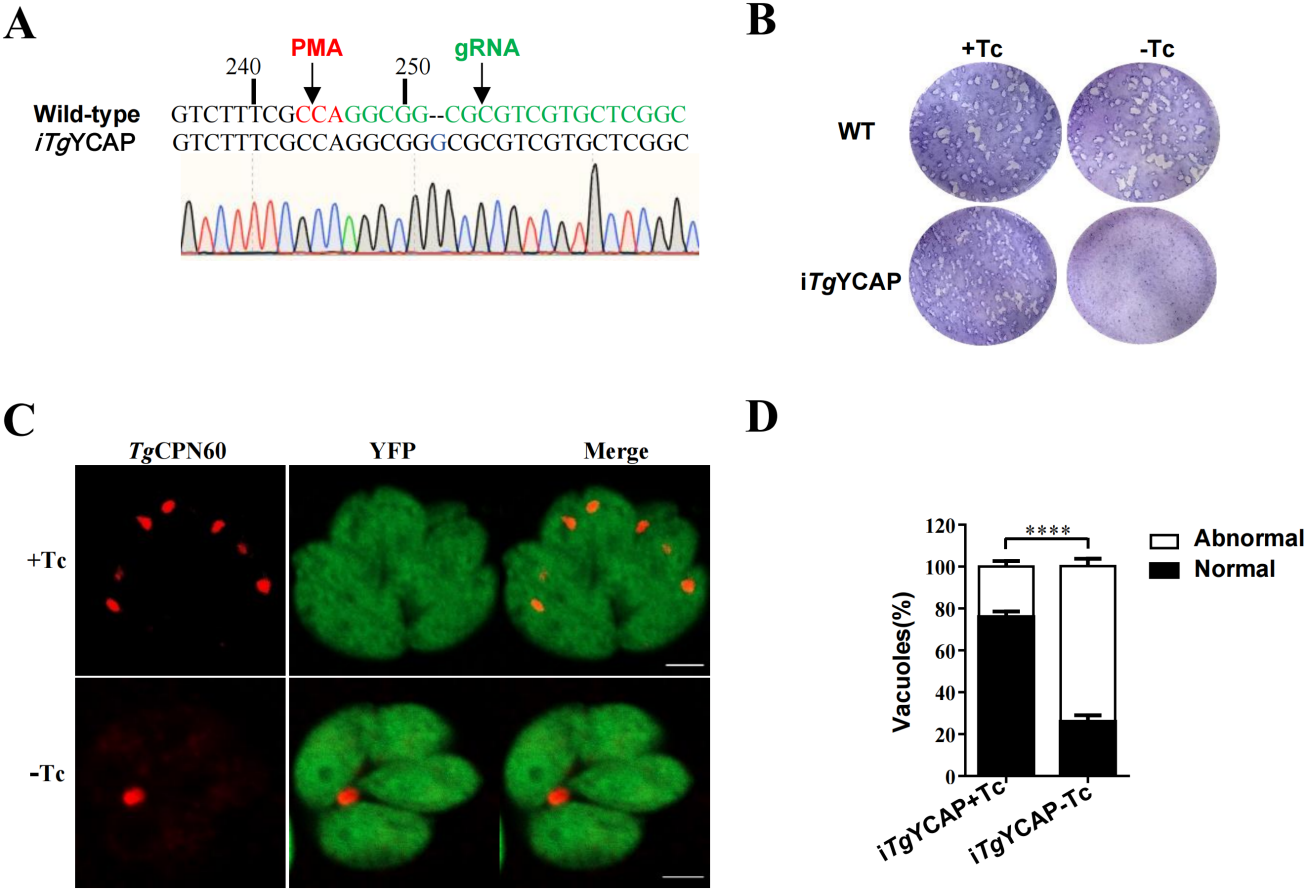
Phenotypic analysis of parasites with conditional deletion of *Tg*YCAP. **(A)** Sequence analysis of the endogenous locus of TgYCAP in the conditional Knockout strain. The inserted base pair was indicated by an arrow. **(B)** Plaque assay. Parasites were cultured in the presence or absence of Tc for 8 days. **(C)** Representative images showing disappearance of the apicoplast in parasites cultured with or without Tc for 3 d. The apicoplast was detected by staining with an antibody against *Tg*CPN60, which is a marker protein of the apicoplast. **(D)** The normal and abnormal PVs in E were counted. At least 100 vacuoles were scored in each condition. All results are the mean of three independent experiments. Error bars indicate the standard error of the mean. ****P < 0.0001.

To assess the importance of *Tg*YCAP for parasite survival, we employed the CRISPR/Cas9 method to knockout the endogenous *Tg*YCAP in the above-mentioned parasite line expressing *Tg*YCAP under control of Tc. We screened individual clones using sequencing analysis of the region flanking the target site of the guide RNA. One clone had a base pair inserted at position 252 of *Tg*YCAP and was chosen for further analysis (**Figure 4A**). To evaluate whether downregulation of *Tg*YCAP affected parasite growth, we conducted a plaque assay. In the presence of Tc, the parasites formed normal plaques after 8 days, comparable to the background strain. However, no plaques were observed when the parasites were cultured without Tc (**Figure 4B**). This finding indicates that *Tg*YCAP is essential for the viability of *T. gondii*.

### *Tg*YCAP is required for the inheritance of the apicoplast

The localization of *Tg*YCAP in the apicoplast led us to explore its role in apicoplast biogenesis. We cultured parasites for three days without Tc and then used an apicoplast lumen protein, *Tg*CPN60, to assess the inheritance of the apicoplast. In this experiment, parasitophorous vacuoles (PVs) in which all parasites were positively stained for *Tg*CPN60 were classified as normal. Conversely, PVs with at least one parasite that was not stained for *Tg*CPN60 were classified as abnormal. We counted a total of 100 PVs containing four parasites each and compared these with the control group. We found that apicoplast loss occurred significantly more often in *Tg*YCAP-deficient parasites than in the control group (**Figures 4C, D**).

### The YGGT domain is essential for the function of *Tg*YCAP in the inheritance of the apicoplast

**Figure 5:**
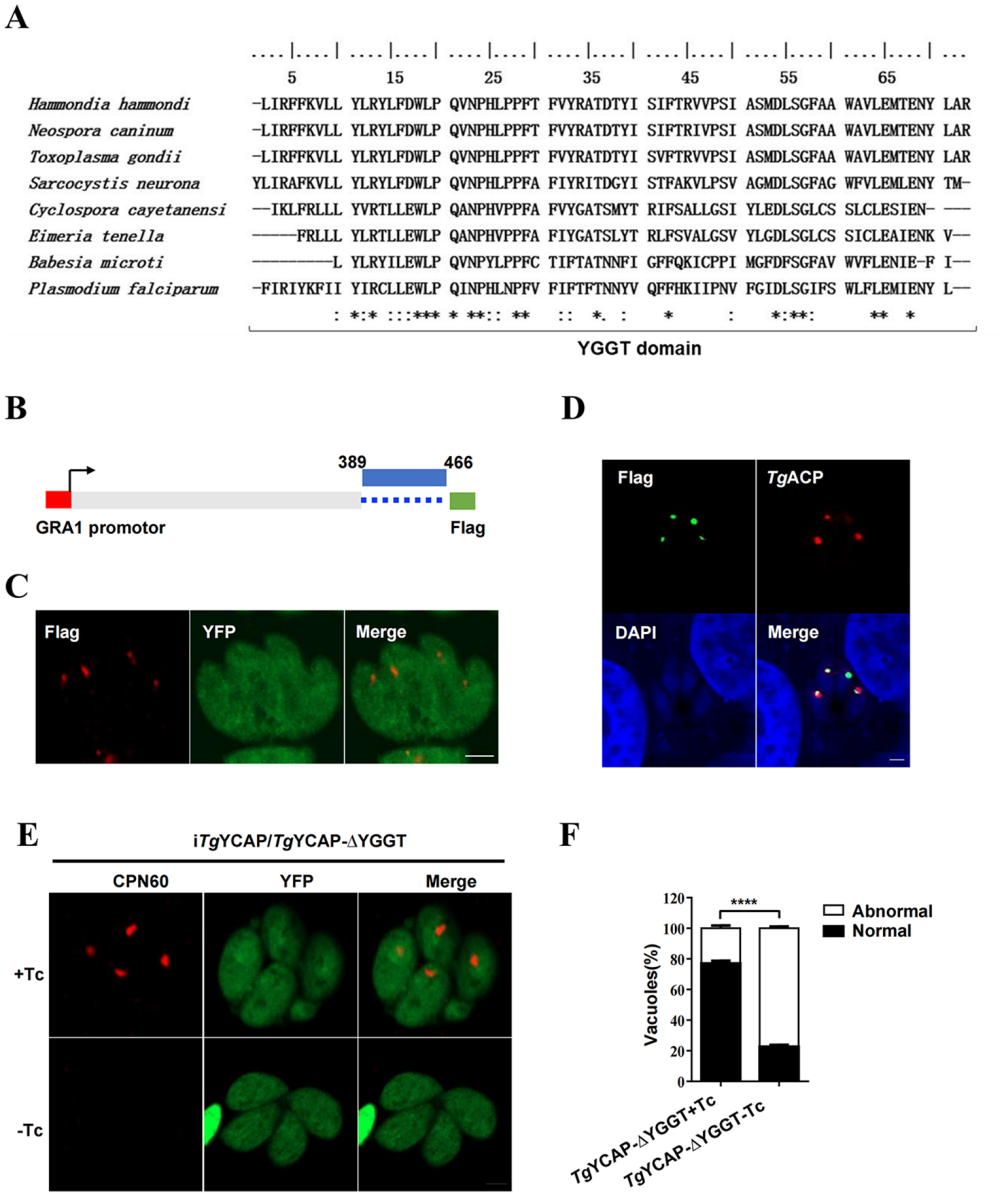
*Tg*YCAP is involved in apicoplast maintenance. **(A)** Multiple alignment of the YGGT domains from different orthologues. The Gap characters "-" are used to indicate the positions of gaps in the multiple alignment. '*' indicates positions which have a single, fully conserved residue. ':' indicates that one of the following 'strong' groups is fully conserved. '.' indicates that one of the following 'weaker' groups is fully conserved. **(B)** A diagram of the construct expressing TgYCAP, which lacks the YGGT domain. **(C)** Localization of the TgYCAP mutant that is missing the YGGT sequence (shown in red). The iTgYCAP parasites were transfected with the pHX-GRA-YCAPΔYGGT plasmid, selected using mycophenolic acid and xanthine, and subsequently stained with a anti-Flag mAb. **(D)** Co-localization of *Tg*YCAPDΔYGGT with the apicoplast marker. Parasites were transiently co-transfected with the above plasmid expressing *Tg*YCAPDΔYGGT and the plasmid expressing TgACP-mcherry(red) and stained with a rabbit anti-Flag antibody (green). **(E)** The maintenance of the apicoplast was monitored. The (iTgYCAP-ΔYGGT) parasites were cultured without tetracycline (Tc) for 3 days and then stained with an anti-TgCPN60 antibody. **(F)** At least 100 vacuoles were scored for each condition from **(D)** All results represent the mean of three independent experiments. Error bars indicate the standard error of the mean. ****P < 0.0001.

The YGGT domain is highly conserved in the Apicomplexa (**Figure 5A**). To investigate the function of this conserved YGGT domain, a mutant lacking this sequence was cloned into the pHX-GRA vector (Cao et al., 2020) (**Figure 5B**) and subsequently transfected into the i*Tg*YCAP strain. IFAT analysis indicated that the recombinant *Tg*YCAP-ΔYGGT-Flag was expressed in the parasites and correctly localized to the apicoplast (**Figures 5C, D**). We monitored the inheritance of the apicoplast both in the presence and absence of Tc. The results from the IFAT demonstrated that the YGGT domain is essential for the function of *Tg*YCAP in apicoplast inheritance (**Figures 5E, F**).

## Discussion

The apicoplast has attracted considerable attention due to its essential role in several metabolic pathways. However, its biogenesis and maintenance are not well understood. Information about the composition of the four apicoplast membranes are lacking, except about their transposons and transporters (van Dooren et al., 2008; Agrawal et al., 2009; Brooks et al., 2010; Glaser et al., 2012; Agrawal et al., 2013; Sheiner et al., 2015; Amberg-Johnson et al., 2017; Fellows et al., 2017). Therefore, more apicoplast membrane proteins must be explored to better understand the biological characteristics of these membranes.

The transport mechanisms that mediate the targeting of nucleus-encoded apicoplast-targeted (NEAT) proteins to the apicoplast are not well understood. Most of these proteins have a clear bipartite N-terminal region, consisting of a signal peptide (SP) followed by an apicoplast transit peptide (TP). This bipartite region is generally sufficient for delivering the proteins to the apicoplast. However, some NEAT proteins, such as TgAPT1 (DeRocher et al., 2012), TgATrx1, and TgATrx2 (Prasad et al., 2021), do not exhibit an obvious TP and/or SP. Although the N-terminal region (aa 14 to 38) of TgAPT1 is critical for its transport to the apicoplast, the N-terminal alone is not enough to deliver a fusion protein to the apicoplast (DeRocher et al., 2012). Therefore, different mechanisms likely exist for transporting these NEAT proteins. In the case of APT1, a YG motif facilitates its trafficking to the apicoplast. Bioinformatic analyses have also failed to identify a clear signal peptide in TgYCAP. We found that amino acids 1–200 are important for the transport of TgYCAP to the apicoplast. It will be interesting to investigate whether a similar YG motif exists in the N-terminal region of TgYCAP.

The function of YGGT family proteins is largely unknown. A specific subgroup of YGGT family proteins is clearly associated with the maturation of c-type cytochromes, as exemplified by CCB3 from *Chlamydomonas reinhardtii* (Lezhneva et al., 2008). This function is expected to be unuseful for non-photosynthetic eukaryotes and bacteria. Nonetheless, two key points should be highlighted to further clarify the function of *Tg*YCAP. First, a recent study suggests that YlmG may create a potential translocation path for TIC (translocon at the inner chloroplast membrane) complexes with its two transmembrane helices along with the six helices of Tic20, although there is currently no functional or biochemical evidence to support this structural finding (Jin et al., 2022; Liu et al., 2023; Liang et al., 2024). Our data indicate that the knockdown of *Tg*YCAP significantly disrupts parasite growth. Additionally, while the YGGT domain is not necessary for the proper localization of *Tg*YCAP, it is crucial for its role in the inheritance of the apicoplast. Bioinformatic analysis has shown that the YGGT domain of *Tg*YCAP also contains two α-helices. Therefore, it is valuable to investigate whether apicoplasts employ a this mechanism for importing NEAT proteins.

Second, ftsZ of bacteria is a tubulin homolog and form the Z-ring together with its membrane anchors to initiate the assembly of the divisome during the cell division process. The ylmG gene in gram-positive bacteria is located within the cell division gene cluster, downstream of ftsZ. Furthermore, the YlmG protein from *Arabidopsis thaliana* (AtYLMG1-1) has been found to play a significant role in the distribution of nucleoids within chloroplasts (Kabeya et al, 2010). Overexpression of AtYLMG1–1 influences the division of chloroplasts. Therefore, it is reasonable to speculate that the function of YGGT proteins may be conserved in the division processes of both plastids and bacteria. Further investigation of the function of *Tg*YCAP in the division process might also enhance our understanding of the molecular mechanism of apicoplast division.

Our results indicate that the YGGT domain is not necessary for trafficking to the apicoplast, but it is essential for the domain’s function within the organelle. This domain contains α helices that may be involved in importing NEAT proteins, as mentioned earlier. Additionally, transmembrane helices are recognized by a P5A-type ATPase (McKenna et al., 2020; Li et al., 2024), which removes a variety of transmembrane proteins from the ER membrane. This process facilitates the proper targeting of these proteins to other membranes. Therefore, the transmembrane helices of *Tg*YCAP may play a role in its trafficking within the apicoplast.

## Data Availability

The original contributions presented in the study are included in the article/**Supplementary Material**. Further inquiries can be directed to the corresponding author.

## Supplementary material

Supplementary Figure 1
**(A)** A diagram showing the position of the diagnostic PCRs. **(B)** PCR identification for the proper insertion of the 3×HA tag at the **(C)** terminal of *Tg*YCAP.
