# Supplementary figures and images for: A novel YGGT family protein is localized in the apicoplast and is essential for the organelle inheritance
Source: Front Cell Infect Microbiol. 2025 Aug 4;15:1642716. doi: 10.3389/fcimb.2025.1642716 (PMC12358420; doi:10.3389/fcimb.2025.1642716)

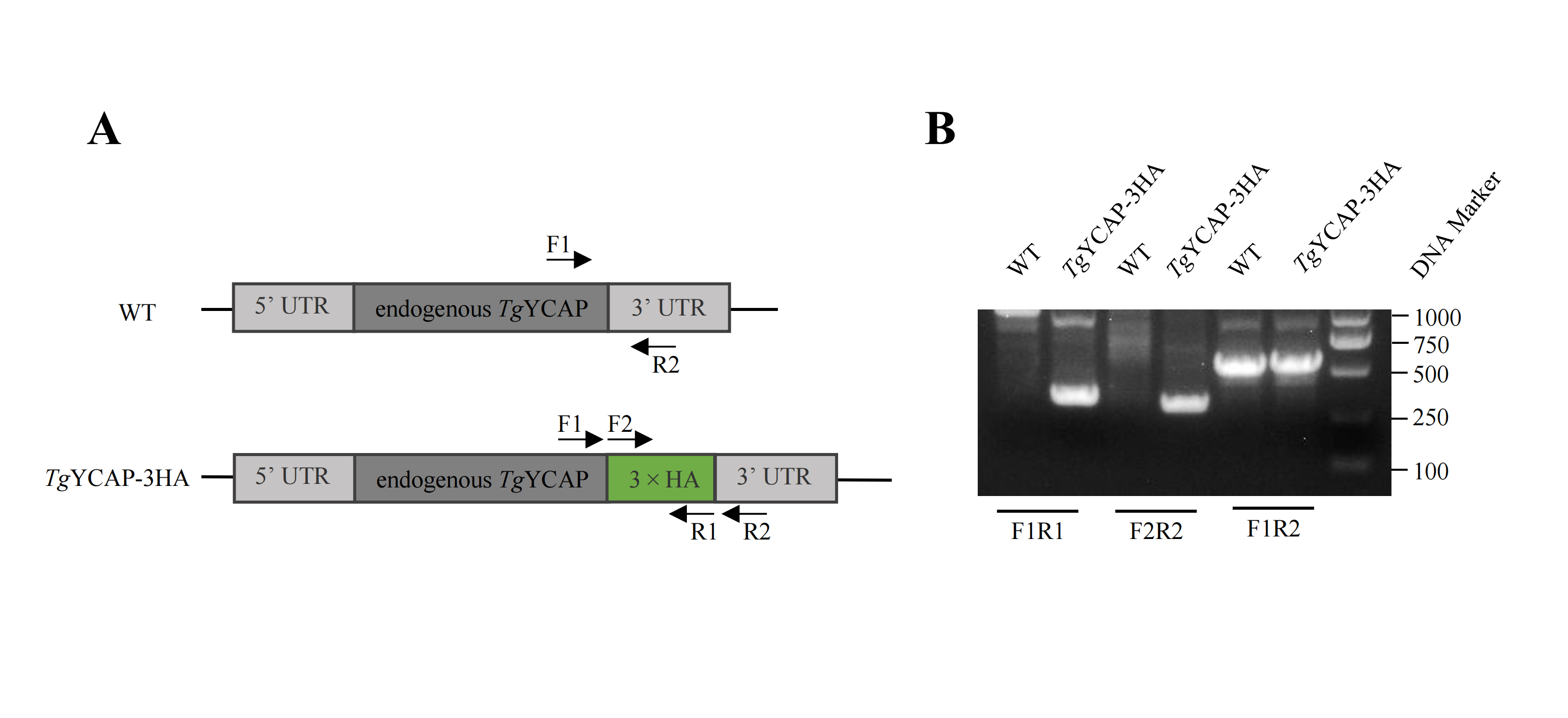

Supplement: Supplementary Figure 1 — (A) A diagram showing the position of the diagnostic PCRs. (B) PCR identification for the proper insertion of the 3×HA tag at the (C) terminal of TgYCAP. [file Image1.tif]
